# Outer Membrane Vesicles, Lipidome, and Biofilm Formation in the Endophyte *Enterobacter Cloacae* SEA01 from *Agave Tequilana*

**DOI:** 10.3390/microorganisms13112432

**Published:** 2025-10-23

**Authors:** Kátia R. Prieto, Hellen P. Valério, Adriano B. Chaves-Filho, Marcos Y. Yoshinaga, Sayuri Miyamoto, Fernanda M. Prado, Itzel Zaizar-Castañeda, Paul Montaño-Silva, América Martinez-Rodriguez, Mario Curiel, Marisa H. G. Medeiros, Flavia V. Winck, Paolo Di Mascio, Miguel J. Beltran-Garcia

**Affiliations:** 1 Department of Biochemistry, Instituto de Química, Universidade de São Paulo, São Paulo 05508-000, SP, Brazil; katia.r.prieto@gmail.com (K.R.P.); hellenpvalerio@gmail.com (H.P.V.); adrianobcfilho@gmail.com (A.B.C.-F.); marcosyukio@gmail.com (M.Y.Y.); miyamoto@iq.usp.br (S.M.); fmprado@iq.usp.br (F.M.P.); mhgdmede@iq.usp.br (M.H.G.M.); 2 Department of Biotecnological and Environmental Sciences, Universidad Autónoma de Guadalajara, Zapopan 45129, Jalisco, Mexico; itzel_iazc97@hotmail.com (I.Z.-C.); paul.montano@edu.uag.mx (P.M.-S.); america.martinez@edu.uag.mx (A.M.-R.); 3 Enginering Institute, Universidad Autonoma de Baja California, Mexicali 21280, Baja California, Mexico; mcuriel@uabc.edu.mx; 4 Laboratory of Regulatory Systems Biology, Center for Nuclear Energy in Agriculture, University of São Paulo, São Dimas, Piracicaba 13416-000, SP, Brazil; winck@cena.usp.br

**Keywords:** *Agave tequilana*, biofilm, catalase-negative, endophyte, *Enterobacter cloacae*, lipidome remodeling, outer membrane vesicle, plant growth promotion

## Abstract

Bacterial outer-membrane vesicles (OMVs) mediate stress tolerance, biofilm formation, and interkingdom communication, but their role in beneficial endophytes remains underexplored. We isolated 11 non-redundant isolates associated with *Bacillus*, *Enterococcus*, *Kosakonia* and *Kocuria* from *Agave tequilana* seeds, identified by MALDI-TOF MS and 16S rRNA gene sequencing. We focused on the catalase-negative *Enterobacter cloacae* SEA01, which exhibits plant-promoting traits and support agave growth under nutrient-poor microcosms. In addition, this endophyte produces OMVs. Time-resolved SEM documented OMV release and cell aggregation within 9 h, followed by mature biofilms at 24 h with continued vesiculation. Purified OMVs (≈80–300 nm) contained extracellular DNA and were characterized by dynamic light scattering and UHPLC–ESI–QTOF-MS lipidomics. The OMV lipidome was dominated by phosphatidylethanolamine (~80%) and was enriched in monounsaturated fatty acids (16:1, 18:1), while the stress-associated cyclopropane fatty acids (17:1, 19:1) were comparatively retained in the whole-cell membranes; OMVs also exhibited reduced ubiquinone-8. SEA01 is catalase-negative, uncommon among plant-associated *Enterobacter*, suggesting a testable model in which oxidative factors modulate OMV output and biofilm assembly. These may have implications for recognition and redox signaling at the root interface. Future works should combine targeted proteomics/genomics with genetic or chemical disruption of catalase/OMV pathways.

## 1. Introduction

Bacteria have developed a variety of adaptation mechanisms that enable them to survive in different and often hostile environments [1]. The formation of biofilms and the release of outer membrane vesicles (OMVs) have been shown to be crucial for microbial communication, stress tolerance and host interactions [2,3,4]. Bacterial OMVs—nanoscale, membrane-bound particles ranging in size from 20 to 400 nm—are produced by both Gram-negative and Gram-positive bacteria [5,6]. Current models of OMV biogenesis include (i) outer membrane blebbing, triggered by an imbalance in the envelope (periplasmic compaction, weakened OM-peptidoglycan bonds, LPS remodeling), and (ii) outer-internal membrane vesicles (OIMVs), which are formed after phage infection, prophage infiltration or endolysin-mediated/explosive lysis and can encapsulate periplasmic and cytoplasmic cargo [7,8,9]. Proteomic and biochemical analyses have shown that OMVs contain many bacterial components, including LPS, outer membrane proteins/lipoproteins, periplasmic enzymes, peptidoglycan, small RNAs and extracellular DNA [10,11]. Their role in pathogenesis and immunomodulation is increasingly recognized [12,13,14]. In addition, OMVs help to alleviate membrane stress by removing misfolded proteins, damaged peptidoglycan and excess lipopolysaccharides, while supporting detoxification and outer membrane remodeling in the presence of nutrient deficiency or oxidative stress [15,16,17].

First observed in *E. coli* in the 1960s [18], bacterial OMVs have only recently attracted attention in the context of plant-associated bacteria [19,20]. For example, in Xylella fastidiosa, a plant pathogen responsible for severe diseases in a number of economically important crops, OMVs reduce cell adhesion in xylem vessels and facilitate systemic movement within the host [19]. Furthermore, OMVs can activate plant immune responses [21]. Such discoveries have led to the conceptualization of bacterial extracellular vesiculation as a type-zero secretion system (T0SS) for selective delivery of bacterial cargoes into the environment [22] such as proteins, peptides, DNA- and RNA-binding proteins along with non-coding small RNAs encoded in genomic islands, suggesting a dual ecological role: enhancing natural bacterial competence and adaptation while supporting host immune defense [23]. Despite recent advances, most studies on bacterial OMVs have focused on pathogens, and their occurrence and function in beneficial plant-associated bacteria, particularly endophytes-remain poorly understood. A few studies have reported OMV production in symbiotic bacteria such as *Rhizobium etli* and *Sinorhizobium fredii* HH103, where flavonoids and isoflavones such as naringenin and genistein induce OMV secretion. These vesicles have been shown to modulate genes, suppress plant defense responses, and influence root development in legumes [24,25,26]. However, research on OMVs from non-rhizobial endophytes, especially those inhabiting seeds or associated with non-leguminous plants, remains scarce.

Endophytes are a diverse group of microorganisms that inhabit healthy plant tissues and are key components of the plant microbiome [27,28]. They can promote growth, enhance stress tolerance, and reduce the need for chemical inputs in agriculture [29,30]. In agave plants, previous studies have shown that seed-borne endophytes contribute to early colonization and activate redox signaling pathways involved in root development and defense responses [31,32,33,34]. Understanding how these microbes colonize plant tissues and communicate with their hosts is essential to fully harness their potential for sustainable agriculture. In this study, we investigated the endophytic strain *Enterobacter cloacae* SEA01 a catalase-negative strain isolated from *Agave tequilana* seeds and selected for its agronomic properties and previous induction of systemic H_2_O_2_ production following colonization of agave roots [35]. Here, we characterize the production of outer membrane vesicles (OMVs) and biofilm formation using scanning electron microscopy (SEM), as well as the lipid composition of both OMVs and bacterial cells. We hypothesize that OMVs and biofilms are complementary strategies used by native endophytes during early plant association. These findings lay the groundwork for future applications in crop microbiome engineering [36].

## 2. Materials and Methods

### 2.1. Seeds and Plant Material

Mature seeds were collected from *Agave tequilana* Weber capsules six months after flowering in 2013. For growth experiments, asexual plantlets (bulbils) were collected from a plantation near Atotonilco el Alto, Jalisco, Mexico (20°34′22.71″ N, 102°32′0.0085″ W; 1900 m a.s.l.). Additionally, commercially micropropagated plants were also obtained from Agromod, Mexico (Tapachula, Chiapas, Mexico).

### 2.2. Isolation of Endophytic Bacteria from A. tequilana Seeds

Agave seeds were surface sterilized by immersion in 3% sodium hypochlorite for 10 min with constant agitation, followed by rinsing with sterile water and immersed in 85% ethanol for 10 min. The seeds were then rinsed three times with sterile water. To confirm successful disinfection, 100 µL of the last rinse water was plated on tryptic soy agar (TSA; BD Bioxon, Naucalpan, Mexico) and incubated at 32 °C for 15 days to monitor microbial growth. Additionally, ten surface-sterilized seeds were also plated directly onto TSA and incubated at 30 °C. The plates were examined daily for colony development. Emerging colonies were isolated, subcultured on fresh TSA plates, and purified. Morphological characteristics were examined by light microscopy and Gram staining was performed.

### 2.3. Identification of Bacterial Endophytes by MALDI-TOF MS and 16S rRNA Gene Sequencing

Bacterial strains were identified by matrix-assisted laser desorption/ionization time-of-flight mass spectrometry (MALDI-TOF MS) using an Autoflex Speed instrument (Bruker Daltonics, Bremen, Germany). Colonies were grown on tryptic soy agar (TSA) at 30 °C for 18 h and processed according to the manufacturer’s protocol. Mass spectra were acquired in linear positive mode over a mass range of 5–20 kDa, using a 35% laser setting and 2000 laser shots per sample. Identification was carried out with MBT Compass Biotyper software 4.1.100 (Bruker; Bremen, Germany), using a reference spectral database. External calibration was performed using *Escherichia coli* DH5α as the standard.

For molecular identification, genomic DNA was extracted from overnight cultures, and the 16S rRNA gene was amplified using universal primers 27F (5′-AGAGTTTGATYMTGGCTCAG-3′) and 1525R (5′-GGYTACCTTGTTACGACTT-3′). PCR was performed with an initial denaturation at 95 °C for 5 min, followed by 30 cycles of denaturation at 95 °C for 30 s, 55 °C for 30 s, 72 °C for 2 min, with a final extension step at 72 °C for 10 min. Amplicons were verified by agarose gel electrophoresis and sequenced using a 3130 Series Genetic Analyzer (IPICYT, San Luis Potosí, Mexico). Sequences were edited and aligned using Geneious 8.1, then compared to entries in the NCBI GenBank database using BLAST software v2.16.0 for taxonomic identification.

### 2.4. Phylogenetic Analysis

Sequences were aligned using ClustalX 2.0 [37] with default parameters (gap opening = 10, gap extension = 1, divergence delay = 30%, and BLOSUM matrix). Alignments were visualized and edited in JalView Version 2 [38]. Phylogenetic analysis was conducted in MEGA11 [39] using the Neighbor-Joining (NJ) with 1000 bootstrap replicates to assess branch support [40]. Evolutionary distances were calculated using the Jukes-Cantor substitution model.

### 2.5. Putative Plant Growth-Promoting Traits: Nitrogen Fixation, Phosphate Solubilization, IAA Production, Siderophore Secretion, and ACC Deaminase Activity

(a) Nitrogen Fixation

The potential nitrogen-fixing ability of strain SEA01 was assessed by culturing it in nitrogen-free semi-solid Nfb-malate medium, as described by Döbereiner et al. [41]. To confirm its ability to fix atmospheric nitrogen, the strain was transferred seven consecutive transfers in Nfb medium.

(b) Indole-3-Acetic Acid (IAA) Production

Indole-3-acetic acid (IAA) production was assessed following the method described by Ullah et al. [42]. Bacteria were cultured for 48 h in tryptic soy broth supplemented with 0.1% L-tryptophan. One milliliter of the centrifuged supernatant was mixed with 2 mL of Salkowski reagent and incubated in the dark for 30 min. The appearance of a pink coloration indicated IAA production.

(c) Phosphate Solubilization

Phosphate solubilization was tested using the plate assay method on Pikovskaya’s agar [43] containing insoluble phosphate sources such as tricalcium phosphate and hydroxyapatite. Spot-inoculated plates were incubated, and the formation of a clear halo around bacterial colonies was considered a positive result.

(d) ACC Deaminase Activity

The ability to produce 1-aminocyclopropane-1-carboxylate (ACC) deaminase was determined using Dworkin and Foster (DF) minimal salt medium supplemented with ACC as the sole nitrogen source [44]. Growth on DF medium was considered indicative of endogenous ACC deaminase activity.

(e) Siderophore Production

Siderophore production was detected using the chrome azurol S (CAS) agar assay [45]. Bacterial colonies were streaked on nutrient agar plates containing 10% CAS reagent and incubated at 30 °C for one week. The appearance of a yellow-to-pink halo around the colonies indicated positive siderophore production.

### 2.6. Catalase Activity and Native PAGE Electrophoresis

Catalase activity was evaluated by placing a loopful of 16 h colony growth onto a clean glass slide and adding a drop of 30% hydrogen peroxide (H_2_O_2_). The immediate formation of oxygen bubbles indicated catalase activity. For protein-level analysis, native PAGE was performed. Crude protein extracts were loaded onto an 8% non-denaturing polyacrylamide gel and electrophoresed at 150 V for 2 h. After electrophoresis, the gel was incubated in 5% methanol with shaking for 5 min, then treated with 10 mM H_2_O_2_ for 10 min. Subsequently, the gel was transferred to a ferricyanide-ferric chloride staining solution (0.3 g potassium ferricyanide in 30 mL distilled water +0.336 g ferric chloride in 30 mL distilled water) for 10 min, and finally incubated in 10% acetic acid for 15 min. Clear bands within a blue background indicated catalase activity, based on H_2_O_2_ degradation by catalase and inhibition of blue pigment formation [46].

### 2.7. OMVs Isolation, Purification, and SEM Visualization

*E. cloacae* SEA01 was cultured in M9 broth adjusted to OD_600_ = 0.2 (approximately 5.3 × 10^5^ CFU mL^−1^) and incubated at 35 °C with agitation (200 rpm). To monitor OMV production, samples were collected at 0, 3, 5, 7, 9, 18, 24, and 48 h. For each time point, 50 mL of culture was centrifuged at 2000× *g* for 20 min at 4 °C. The supernatant was filtered through 0.45 µm and 0.22 µm PVDF membranes (Millipore, Burlington, MA, USA) to remove bacterial cells and debris. OMVs were pelleted by ultracentrifugation at 150,000× *g* for 70 min at 4 °C, resuspended in 200 µL of sterile Milli-Q water containing a protease inhibitor cocktail, and re-filtered through 0.22 µm to ensure sterility. Aliquots (10 µL) of each preparation were plated on TSA and incubated at 37 °C for 5 days to confirm the absence of viable bacteria. Purified OMV suspensions were stored at 4 °C for short-term use (≤1 week) or at –80 °C for long-term preservation.

For scanning electron microscopy (SEM), bacterial pellets were washed three times in 0.08% glucose solution and fixed in 2.5% glutaraldehyde for 1 h. Samples were then washed four times with 0.1 M cacodylate buffer (pH 7.2), post-fixed in 1% osmium tetroxide for 1 h, rinsed again, treated with 1% tannic acid for 30 min, and washed twice with deionized water. A second fixation in 1% osmium tetroxide was carried out for 30 min, followed by triple rinsing with Milli-Q water. Dehydration was performed using a graded ethanol series (10–100%) with 10 min steps. Samples were dried at the critical point and sputter-coated with a 10 nm layer of gold. Observations were conducted using a FEI Quanta FEG 250 scanning electron microscope operated at 5 kV (Thermo Fisher Scientific, Whaltam, MA, USA).

### 2.8. Dynamic Light Scattering (DLS)

Outer membrane vesicles (OMVs) collected at 9 h of culture were used for both DLS and lipidome analyses. Dynamic light scattering (DLS) measurements were performed using a Zetasizer NanoS instrument (Malvern Instruments, Malvern, UK). The average hydrodynamic diameter was calculated from the unimodal size distribution of the vesicles. Samples were diluted 1:500 in 0.2 M NaCl and filtered through a 0.45 µm pore-size membrane to remove aggregates. Measurements were conducted at room temperature, with 40–50 runs per sample over a total duration of 30 min. The intensity-weighted average diameter was recorded, and size values represent the mean of three independent OMV preparations.

### 2.9. Lipid Extraction and Lipidomic Analysis

Lipid extraction was performed following the protocol described by [47]. Briefly, 50 μL of bacterial cells or vesicles were mixed with 450 μL of sodium phosphate buffer (pH 7.4) and 400 μL of ice-cold methanol. Then, 1.5 mL of chloroform/ethyl acetate (4:1, *v*/*v*) was added, and the mixture was vortexed for 30 s. After centrifugation at 1500× *g* for 2 min at 4 °C, the lower organic phase containing the total lipid extract (TLE) was collected and evaporated under a stream of nitrogen gas. The dried TLE was redissolved in 100 μL of isopropanol and spiked with a mixture of external standards for semi-quantification (Table S1). A 1 μL aliquot was injected for analysis.

Lipid profiling was conducted using electrospray ionization time-of-flight mass spectrometry (ESI-TOFMS; Triple TOF 6600, Sciex, Concord, Framingham, MA, USA) coupled to an ultra-high performance liquid chromatography system (UHPLC Nexera, Shimadzu, Kyoto, Japan), as previously described [48]. The MS was operated in negative ionization mode with a scan range of *m*/*z* 200–2000. Data acquisition was performed using Information-Dependent Acquisition (IDA^®^) mode. Lipid identification was based on retention time, accurate mass, characteristic fragment ions and/or neutral losses [49] using an internally developed Excel-based macro. Peak areas were quantified with MultiQuant^®^ software 3.0.3, and the area ratios were calculated by normalizing to the external standard. The relative abundance (%) of each lipid species was determined within its corresponding lipid class.

### 2.10. Statistical Analysis

The data were analyzed using SPSS 22.0 (IBM). A one-way ANOVA was performed to determine significant differences between treatments in plant dry biomass accumulation (Figure S3). Values of *p* ≤ 0.05 were considered statistically significant.

## 3. Results

### 3.1. Identification of Bacterial Endophytes from Agave Seeds

A total of 50 bacterial endophytes were isolated from *Agave tequilana* seeds. Isolates were selected based on colony morphology, including differences in size, pigmentation, and appearance time within the first 72 h of incubation. After eliminating redundant morphotypes (sibling colonies), 11 unique isolates were selected and identified using matrix-assisted laser desorption/ionization–time of flight (MALDI-TOF) mass spectrometry. These isolates were assigned to the genera *Bacillus* (*B. aerius*, *B. altitudinis*, *B. megaterium*, *B. tequilensis*, *B. safensis*, *B. subtilis*, and *B. pumilus*), *Enterococcus casseliflavus*, *Enterobacter cloacae*, *Kosakonia cowanii*, and the actinobacterial genus *Kocuria* (*K. marina*). To validate the identifications, 16S rDNA sequencing was performed on all selected isolates, revealing a 95% concordance with the MALDI-TOF results. Notably, the strain identified as *Bacillus megaterium* by MALDI-TOF was classified as *Priestia megaterium* based on 16S rDNA, consistent with the reclassification of this species [50]. The 16S rDNA sequences were deposited in GenBank under the following accession numbers: *E. cloacae* SEA01 (KY625189.1), *E. casseliflavus* x19 (MF322527.1), *K. cowanii* Agave3 (KY681445.1), *K. marina* KM (KY681446.1), *B. altitudinis* A6-2111 (MF567399.1), *B. pumilus* A12-212 (MF567388.1), *B. safensis* ATC9 (KY476353.1), *B. tequilensis* 29G (MF540451.1), *B. aerius* PMBG1 (MF288782.1), *B. subtilis* Rb (MF322532.1), and *P. megaterium* B511-5 (MF322535.1).

**Figure 1:**
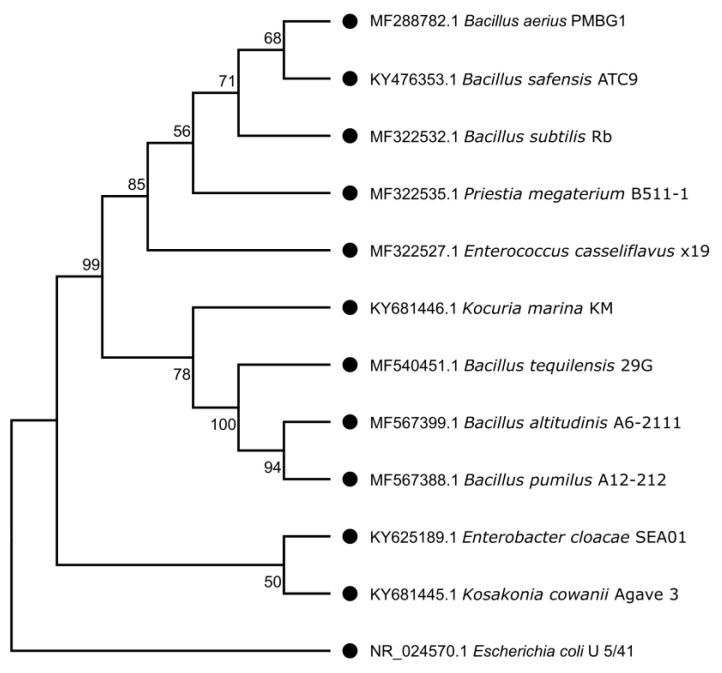
Phylogenetic relationships of endophytic bacteria isolated from *Agave tequilana* seeds based on 16S rRNA gene sequences. The phylogenetic tree was constructed using the Neighbor-Joining (NJ) method with *E. coli* U5/41 16S (NR_024570) [51] as an outgroup. Bootstrap values (1000 replicates) are shown at the nodes to indicate the reliability of the branching. The tree highlights the taxonomic position of isolates within the phyla *Bacillota*, *Pseudomonadota*, and *Actinomycetota*, confirming the diversity of endophytic bacteria colonizing *A. tequilana* seeds.

A phylogenetic tree based on the Neighbor-Joining method was constructed to illustrate the evolutionary relationships of the isolates (Figure 1). Most isolates belonged to the phylum *Bacillota*, distributed among the orders *Bacillales*, *Caryophanales*, and *Lactobacillales* (7 isolates), followed by 2 isolates in the phylum *Pseudomonadota* (order *Gammaproteobacteria*), and one isolate from the phylum *Actinomycetota* (order *Micrococcales*).

### 3.2. Qualitative Characterization of Agave Endophytes as Plant Growth-Promoting Bacteria (PGPB)

Table 1 summarizes the plant growth-promoting (PGP) traits of seven endophytic bacteria isolated from *A. tequilana* seeds, based on qualitative and microbiological assessments. Approximately 70% of the isolates exhibited nitrogen fixation activity, except for *B. safensis*, *B. aerius* and *B. altitudinis*. Regarding phosphate solubilization, 63% of the isolates were positive. The production of auxin, particularly indole-3-acetic acid (IAA)-like compounds, was detected in *B. tequilensis*, *K. marina* and *E. casseliflavus*. Regarding the production, siderophores was observed in 54% of the strains. Remarkably, only 45% of the isolates demonstrated 1-aminocyclopropane-1-carboxylate (ACC) deaminase activity a key property associated with alleviating plant stress by degrading the ethylene precursor ACC, thereby reducing ethylene-induced senescence. Among the isolates, *E. cloacae* and *B. pumilus* exhibited the most plant growth-promoting properties, although neither produced auxins, as shown in Table 1.

### 3.3. Visualization of OMVs Release and Biofilm Formation by E. cloacae SEA01

Previous studies have shown that *E. cloacae* modulate endophytic root colonization [52,53,54]. It has been demonstrated that, upon perceiving bacterial presence, plants secrete hydrogen peroxide to eliminate or restrict microbial access [55,56]. Therefore, the ability to form biofilms confers a survival and colonization advantage to *Enterobacter* strains, as has already been reported for some isolates [57,58,59,60].

**Figure 2:**
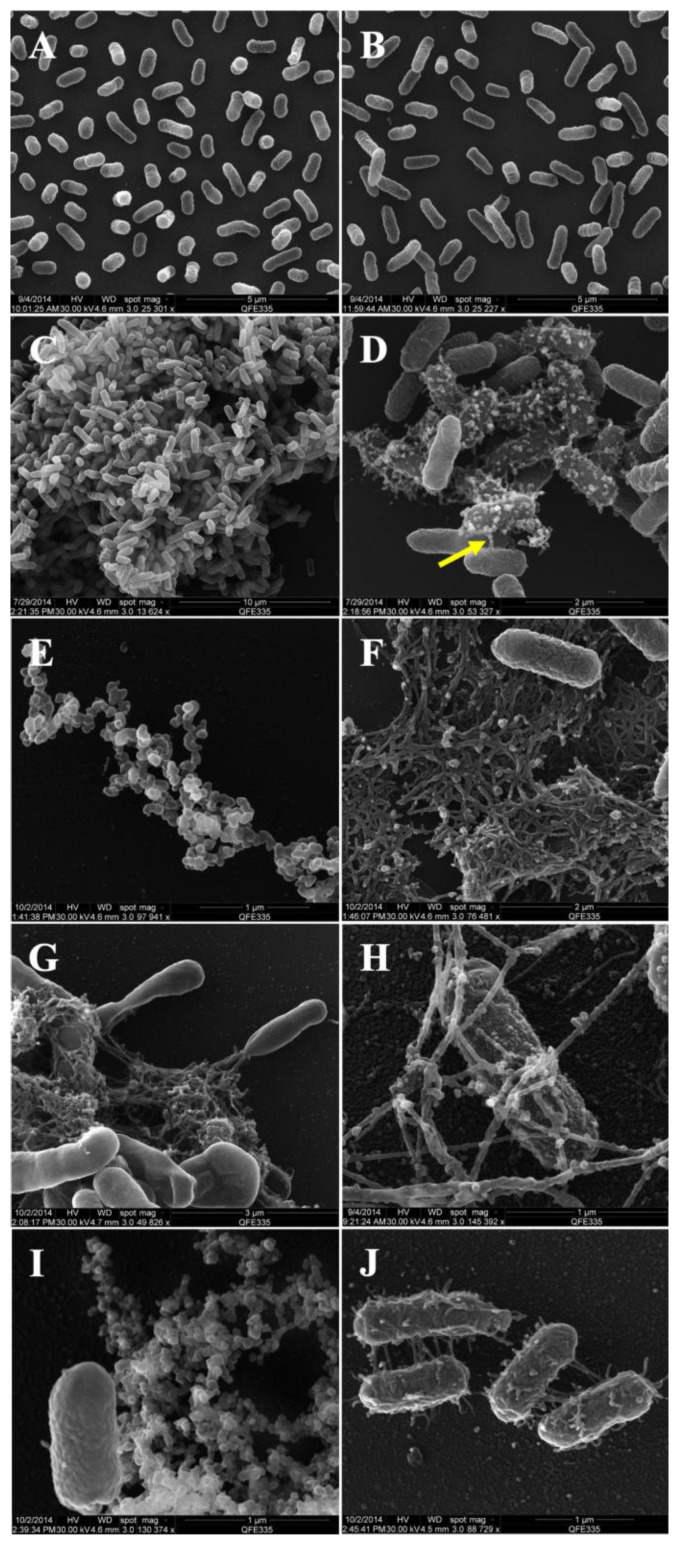
Scanning electron micrographs illustrating the dynamic process of outer membrane vesicle (OMV) production and biofilm formation by *E. cloacae* SEA01 over a 48 h period. (**A**) At 0 h, bacterial cells exhibit typical rod-shaped morphology. (**B**) At 5 h, elongation of cells indicates active division in liquid M9 medium. (**C**) At 9 h, microcolony formation begins, accompanied by initial vesicle production on the cell surface. (**D**) Close-up view of OMVs and intercellular connections via “nanopod”-like projections (yellow arrow). (**E**) At 18 h, detached OMVs are observed dispersed in the biofilm matrix. (**F**) Aggregated biofilm-derived extracellular vesicles (OMVs) embedded in a maturing biofilm matrix at 18 h. (**G**) At 24 h, a mature biofilm structure with actively migrating cells is evident. (**H**) Filamentous networks, possibly pili, are seen connecting cells and are coated with OMVs. (**I**) After 48 h, extensive vesicle OMV release occurs and (**J**) Cells at the late stage are densely covered fimbriae-associated OMVs. Scale bars are indicated in each panel.

Scanning electron microscopy (SEM) images reveal the dynamics of biofilm formation and outer membrane vesicle (OMV) production by *Enterobacter cloacae* SEA01over a 48 h time course (Figure 2). At the initial time point (0 h), bacterial cells exhibited a typical rod-shaped morphology (~0.3 × 0.5 μm), with smooth surfaces and uniform distribution (Figure 2A). After 5 h (Figure 2B), cells elongation was observed, suggesting active division and early exponential growth, while 9 h, dense microcolonies had formed (Figure 2C), and numerous extracellular vesicles were visible on the bacterial surface and within the surrounding matrix (Figure 2D). Notably, thin projections or “nanopods” (indicated by yellow arrow) were seen connecting neighboring cells and suggesting long-distance interaction.

At 18 h, extracellular vesicles appeared aggregated into organized chains and clusters (Figure 2E), coinciding with the early development of the biofilm matrix (Figure 2F). The emerging biofilm displayed a complex reticular architecture composed of vesicles and filamentous components. After 24 h (Figure 2G–H), a mature biofilm structure was visible, characterized by dense bacterial aggregates interconnected by filamentous structures, along with signs of bacteria migration away from the matrix. These filamentous structures may correspond to pili or extracellular fibers involved in intercellular communication and vesicle anchoring. OMVs were observed adhered to these filaments (Figure 2H), indicating a potential structural or signaling role.

At 48 h (Figure 2I–J), the number of most bacterial cells had decreased, but vesicle release persisted. Some cells had large amounts of vesicles on their surface and fimbria-like extensions, indicating a final phase of vesiculation and possible late-stage biofilm remodeling (Figure 2J). These observations confirm the ability of *E. cloacae* SEA01 to produce OMVs in a growth stage-dependent manner and to form structured biofilms, both of which may contribute to its plant-associated lifestyle and plant growth-promoting activities.

### 3.4. Characterization of DLS and Zeta Potential

**Figure 3:**
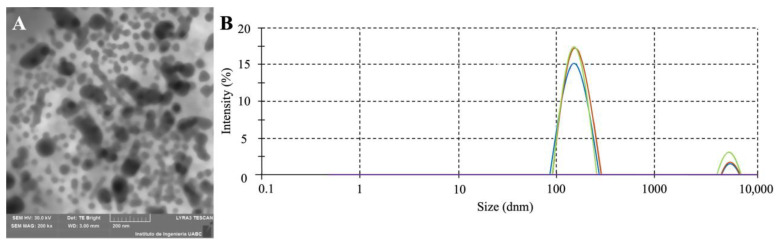
Characterization of outer membrane vesicles (OMVs) isolated from *E. cloacae* SEA01. (**A**) Scanning electron micrograph showing spherical OMVs of varying sizes, characterized by a double-membrane structure and electron-dense filamentous material within the lumen. (**B**) Dynamic light scattering (DLS) analysis indicating a unimodal size distribution of OMVs ranging from 75 to 400 nm, with an average hydrodynamic diameter of 153.1 nm. Each colored line (green, blue, orange) denotes an independent OMV sample/preparation measured under identical conditions.

Dynamic light scattering (DLS) analysis was conducted on vesicles isolated from a 9 h culture of *E. cloacae* SEA01 (Figure 3A). The DLS results revealed a unimodal size distribution ranging from 75 to 400 nm, with an average hydrodynamic diameter of 153.1 nm (Figure 3B). To assess the colloidal stability of the vesicle suspensions, Zeta potential measurements were also performed. Typically, absolute Zeta potential values exceeding ±30 mV are indicative of stable dispersions due to strong electrostatic repulsion, while lower values suggest propensity for particle aggregation. The vesicles of *E. cloacae* SEA01 exhibited an average zeta potential of −26.1 mV and an electrophoretic mobility of −2.041 μm-cm/V-s, suggesting moderate colloidal stability and a tendency toward aggregate.

### 3.5. Lipid Analysis of OMVs

**Figure 4:**
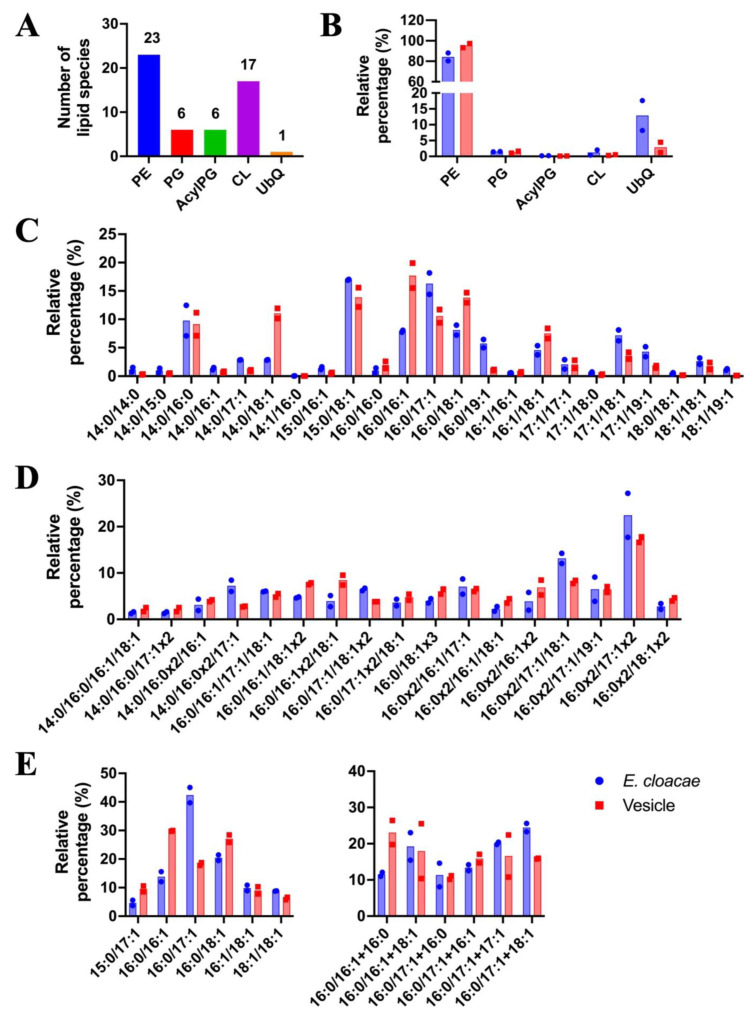
Lipid composition of *E. cloacae* SEA01 vegetative cells and their outer membrane vesicles (OMVs). (**A**) Total number of identified lipid species grouped into five lipid classes: phosphatidylethanolamine (PE), phosphatidylglyceron (PG), acylphosphatidylglycerol (Acyl-PG), cardiolipin (CL), and ubiquinone (UbQ8). (**B**) Relative abundance (5%) of each lipid class in vegetative cells (blue) and OMVs (red), showing PE as the dominant class in both fractions. (**C**–**E**) Relative distribution of individual lipid molecular species within each class (**C**) PE, (**D**) CL, and (**E**) PG and Acyl-PG. OMVs exhibited a enrichment in lipid species containing 16:1 and 18:1 fatty acyl chains-such as PE (16:0/16:1) and PG (16:0/18:1)-while species containing 17:1 and 19:1 chains were more abundant in vegetative cells. Data represent means ± SD of two biological replicates (*n* = 2). Colored bars represent OMVs (red) and vegetative cells (blue).

Lipid profiling of *E. cloacae* SEA01 vegetative cells and OMVs was conducted 9 h post-inoculation using ultra-high-performance liquid chromatography coupled with electrospray ionization time-of-flight mass spectrometry (UHPLC-ESI-TOF-MS). As shown in Figure 4A, a total of 53 distinct lipid species were identified and classified into five major lipid classes: phosphatidylethanolamines (PE; 23 species), phosphatidylglycerols (PG; 6 species), acyl-phosphatidylglycerols (Acyl-PG; 6 species), cardiolipins (CL; 17 species) and ubiquinone-8 (UbQ8; 1 species). Among these, PE dominated the lipid profile, comprising ~80% of the total lipid content in both cells and OMVs. This predominance of PE is consistent with its known structural role in Gram-negative bacterial membranes, where it contributes to membrane curvature and facilitates vesicle formation (Figure 4B).

The analysis of the percentage contribution of each lipid class indicated differing profiles for vegetative cells and OMVs. Fatty acyl compositions were selectively modulated in OMVs, indicating preferential and likely controlled selection of the lipids composing the OMVs. In the PE class (Figure 4C–E), vesicles exhibited a higher proportion of unsaturated species such as PE (16:0/16:1), PE (16:0/18:1) and PE (14:0/18:1), suggesting preferential sorting of these lipids into the vesicles. Conversely, fatty acyl species with 17:1 or 19:1 (e.g., PE (16:0/17:1), PE (16:0/19:1) were underrepresented in OMVs. In the cardiolipin (CL) profile (Figure 4D), OMVs were enriched with species such as CL (16:0/16:0/17:1/17:1) and CL (16:0/16:0/17:1/18:1), further supporting a reorganization of membrane microdomains during vesicle biogenesis. Similarly, OMVs exhibited distinct PG and acyl-PG profiles (Figure 4E), with increased abundance of PG (16:0/16:1), PG (16:0/18:1) and acyl-PG (16:0/18:1) species, which may contribute to membrane curvature and vesicle release.

## 4. Discussion

*Agave tequilana*, the most important species for tequila production, suffers from low genetic diversity due to clonal propagation, which increases both the need for fertilizers and susceptibility to pests and diseases. In this context, endophytic bacteria play an important role by promoting plant growth, nutrient uptake and stress tolerance [29]. Understanding these symbiotic interactions is particularly given the rising global demand for tequila [61]. Here, we isolated endophytic bacteria from mature seeds of A. *tequilana*, including genera such as *Bacillus*, *Enterobacter*, *Enterococcus*, *Kosakonia*, *Kocuria* and *Priestia* (Figure 1). Although these genera are commonly associated with microbiomes and have been previously detected in various agave tissues [32,33], *Enterococcus casseliflavus* remains the only species so far reported as a seed endophyte of *A. tequilana* [62]. Despite the limited number of cultivable isolates obtained, the recovered bacterial community included representatives of the three major phyla typically found in agaves: Firmicutes, Proteobacteria and Actinobacteria [33].

Seed endophytes represent a conserved segment of the plant microbiota and have a significant impact on the initial stages of plant growth and the establishment of the microbial community. To evaluate the plant growth-promoting potential of the isolated strains, we performed a series of functional assays targeting classical plant growth-promoting traits, including atmospheric nitrogen fixation, solubilization of inorganic phosphate, production of indole-3-acetic acid (IAA), secretion of siderophores, and ACC deaminase activity. Our analysis shows that both *B. pumilus* A12-212 and *E.* cloacae SEA01 tested positive for nitrogen fixation, phosphate solubilization, siderophore production and ACC deaminase activity. *B. pumilus* A12-212 is currently being evaluated as part of the development of a bioinoculant formulation for agave and other crops as a biostimulant and for control of fungal pathogens.

Previous work by our group has shown that *E. cloacae* SEA01 induces systemic H_2_O_2_ accumulation in agave seedlings after root inoculation [35]. However, the effects on seedling biomass were not investigated. Here, continuous application of SEA01 for six months in nutrient-poor sand microcosms was shown to increase seedling dry biomass compared to water-irrigated controls (Supplementary Figure S3). This growth-promoting trend could be due in part to rhizophagy-like processes, i.e., internalization of plants and degradation of associated bacteria during colonization [31,63,64]. This phenomenon, termed “rhizophagy” by the James White Group, involves the root-mediated uptake of bacteria through oxidative processes with hydrogen peroxide (H_2_O_2_) [65]. The *Enterobacter* strains have been described as endophytes that can colonize a variety of plant hosts and interact with the microbiome of native plants. Their effective root colonization by E. cloacae has been linked to their competitive ability and their capacity to form biofilms supported by components of the extracellular matrix [55,58,66].

Outer membrane vesicles (OMVs) produced by Gram-negative bacteria play a role in cell-to-cell signaling, biofilm formation and stress responses [8,12,13]. Herein, we describe the release dynamics and structural features of OMVs produced by E. cloacae SEA01. These vesicles exhibit a size distribution of 75–400 nm and show a zeta potential indicating moderate colloidal stability and a tendency to aggregate (Figure 2). Vesicle production begins around the 9th hour of growth, which coincides with the logarithmic phase and early stages of biofilm formation. Notably, nanopod-like structures have been observed connecting the bacterial cells (Figure 2D), possibly facilitating the transport of OMVs and promoting local metabolic interactions within the biofilm microenvironment [67]. As the culture transitions to stationary phase (after 18 h), biofilm formation comes to the fore, with filamentous networks and aggregated OMVs clearly visible in the matrix (Figure 2F). This pattern of OMV production is like the vesicle formation observed in other bacterial species such as *E. cloacae* ATCC 13047 [68], *Ferrividacidithiobacillus caldus* [69], the Vibrio predator *Pseudoalteromonas piscicida* [70] and *Pseudomonas chlororaphis* under abiotic stress conditions [71].

During the first 24 h of culture, all stages of the biofilm life cycle were observed, including the maturation and subsequent spreading of the bacterial cells (Figure 2G). These spreading cells exhibited adherent OMVs on their surface, along with filamentous structures associated with fimbriae, suggesting active OMV deposition. This observation suggests a possible second wave of vesicle release. Even after 48 h, some cells continued to release OMVs despite a decrease in bacterial density. These results emphasize the central role of OMVs in the development of the biofilm matrix and in supporting bacterial persistence under conditions of nutrient limitation and environmental stress. Electrophoretic analysis of intact OMVs and cells of *E. cloacae* SEA01 (Supplementary Figure S1) revealed that the OMVs contain aggregated extracellular DNA (eDNA) and were compared to OMVs of *E. cloacae* C2 strain, a banana endophyte [51]. This eDNA, together with other components of the biofilm matrix, has an important structural function that promotes both bacterial survival under nutrient-poor conditions and intercellular communication [4,72,73,74]. In addition, during biofilm maturation, eDNA also contributes to the degradation of the scaffold and facilitates cell spreading [75], as shown by the bacterial escape observed in Figure 2G.

Comparative lipidomics of purified OMVs and whole cells of *E. cloacae* SEA01 revealed five major classes. Phosphatidylethanolamine (PE) dominated in both profiles (~80%), consistent with its known role as the major phospholipid in Gram-negative bacteria, where it facilitates membrane expansion and vesicle formation (Figure 4B) [76]. Compared to whole cells, OMV tended to accumulate monounsaturated acyl chains (16:1, 18:1) and PE (14:0/18:1, 16:0/16:1, 16:0/18:1), which likely increase membrane flexibility and promote vesicle formation (Figure 4C) [7,77]. A lower abundance of 17:1 and 19:1 fatty acid species was observed in OMVs, possibly corresponding to cyclopropane fatty acids (CPAs), which have the same mass as their monounsaturated fatty acid counterparts. CPAs are widely distributed in bacteria, from *E. coli* to *H. pylori*. Although the functions of CPAs are not yet fully understood, they are associated with stress responses and may be selectively retained in the cell membrane to preserve structural integrity while being excluded from vesicles, consistent with trends toward selective lipid sorting that preserves envelope rigidity under stress [78,79,80,81]. Lower classes (PG and acyl-PG) may support vesicle stabilization and host interactions, while cardiolipin (CL) likely reflects the bacterial response to stress [77]. OMVs also showed a trend towards reduced ubiquinone-8 (Q8) compared to whole cells, consistent with reports that Q8 increases membrane stiffness and Q8 deficiency increases superoxide generation in *E. coli* [82,83,84]. Altogether, these trending remodeling processes support a model in which OMV production accompanies early biofilm development, as has been proposed [85]. However, targeted lipidomics is required to understand how *E. cloacae* SEA01 shapes OMV for interaction with plants as a colonizer endophyte.

Finally, we would like to emphasize that *E. cloacae* SEA01 is a catalase-negative strain (Supplementary Figure S2), an unusual characteristic among plant-associated *Enterobacter*. As far as we are aware, no endophyte has been reported to combine catalase deficiency with OMV production during association with the host. This observation highlights a potential functional role of OMVs in the maintenance of an endophytic lifestyle and makes them a useful tool as secretory system in various environmental situations [86,87,88]. We therefore hypothesize that catalase deficiency triggers OMV biogenesis and early biofilm development, which could provide a physiological advantage at the onset of plant association. Definitive testing will require comparison with catalase-positive closely related to *Enterobacter* endophytes and/or catalase complementation in SEA01, as well as omics analyzes and assays in plants to define the specific roles of OMVs during root interaction, nutrient transfer and colonization of the blue agave.

## 5. Conclusions

This study shows that the agave-seed endophyte *Enterobacter cloacae* SEA01 supports *Agave tequilana* growth in nutrient-poor microcosms and produces outer-membrane vesicles (OMVs) associated with early biofilm development and lipid signature approach consistent with vesiculation. Notably, SEA01 is catalase-negative, an uncommon feature in plant-associated *Enterobacter*, leading to a testable model in which oxidative factors influence OMV output and biofilm assembly. This raises questions about their roles in recognition and redox signaling at the root interface.

As SEA01 belongs to the *E. cloacae* complex containing ESKAPE lineages, any translational application must be preceded by rigorous biosafety characterization (antibiotic susceptibility testing, genome-based AMR/virulence screening). Future works should combine targeted proteomics/genomics with genetic or chemical disruption of catalase/OMV pathways. These results will provide testable opportunities to develop safe OMV-based strategies as cell-free biostimulants for agave and other crops while clarifying the mechanistic role of OMVs during early plant association.

## Figures and Tables

**Table 1:** Qualitative assessment of plant growth-promoting traits in seven representative endophytic bacteria isolated from *Agave tequilana* seeds.

Strain	Nitrogen Fixation	Phosphate Solubilization	IAA	Siderophore	ACC-Deaminase
*E. cloacae* SEA01	+	+	-	+	+
*K. cowanii* Agave 3	+	+	-	+	-
*P. megaterium* B511-1	+	+	-	+	-
*B. altitudinis* A6-2111	-	-	-	+	+
*B. pumilus* A12-212	+	+	-	+	+
*B. aerius* PMBG1	-	-	-	-	+
*B. subtilis* Rb	+	+	-	-	+
*B. tequilensis* 29G	+	+	+	-	-
*B. safensis* ATC9	-	-	-	+	-
*K. marina* KM	+	-	+	-	-
*E. casseliflavus* x19	+	+	+	-	-

## Data Availability

The original contributions presented in this study are included in this article and Supplementary Materials; and all the 16SrRNA gene sequences of the identified endophytic strains are openly available in the National Center for Biotechnology Information (NCBI) data base under accession numbers: *E. cloacae* SEA01 (KY625189.1), *E. casseliflavus* x19 (MF322527.1), *K. cowanii* Agave3 (KY681445.1), *K. marina* KM (KY681446.1), *B. altitudinis* A6-2111 (MF567399.1), *B. pumilus* A12-212 (MF567388.1), *B. safensis* ATC9 (KY476353.1), *B. tequilensis* 29G (MF540451.1), *B. aerius*.

## Supplementary Materials

The following supporting information can be downloaded at: https://www.mdpi.com/article/10.3390/microorganisms13112432/s1. Figure S1: Detection of DNA associated with outer membrane vesicles (OMVs) from Enterobacter cloacae strains SEA01 and C2; Figure S2. Representative native PAGE gel showing in-gel catalase activity; Figure S3. Effect of E. cloacae SEA01 on the total dry biomass of A. tequilana plantlets after six months of treatment; Figure S4. Principal component analysis of lipidomics data from whole Bacteria and bacteria’s Outer membrane vesicles. Table S1. External standards used for the semi quantification in the lipidomic analysis.
