# Supplementary material for: Outer Membrane Vesicles, Lipidome, and Biofilm Formation in the Endophyte Enterobacter Cloacae SEA01 from Agave Tequilana
Source: Microorganisms. 2025 Oct 23;13(11):2432. doi: 10.3390/microorganisms13112432 (PMC12654736; doi:10.3390/microorganisms13112432)
Supplement: Supplementary file 1 [file microorganisms-13-02432-s001.zip › Supplementary material OMVs (1).pdf]

Supplementary Material.

1. **Extraction of chromosomal DNA from SEA01 vegetative cells and electrophoretic analysis of purified OMVs**

Chromosomal DNA extraction from bacterial cells

Chromosomal DNA was extracted from vegetative cells of Enterobacter cloacae SEA01 and *E. clocaae* C2 (a banana endophyte, see [52]). Bacteria culture were grown for 16 hours at 32°C on Petri dishes. A loopful of culture was suspended in 300 µl of Milli-aQ water and subjected to three washing steps, each followed by centrifugation at 12,000 rpm for 5 minutes. The final pellet was resuspended in 250 µl DNAzol® *(Thermo Fisher Scientific)* and 250 µl Tris-EDTA buffer (pH 8.0), vortexed for 5 minutes and centrifuged at 11,000 rpm for 12 minutes. The supernatant was discarded.

The resulting pellet was resuspended in 500 µl of absolute ethanol (molecular biology grade, Sigma-Aldrich), gently mixed, and incubated at −80 °C for 5 minutes. After incubation, samples were centrifuged at 11 000 rpm for 10 minutes, followed by two washes with 500 µl of 70% ethanol, each with centrifugation at 11, 000 rpm for 5 minutes. The final pellet was air-dried at room temperature (5–10 minutes) and resuspended in 20–100 µl of sterile Milli-Q water. DNA concentration and purity were determined using an Eppendorf BioSpectrometer® Basic (hamburg, Germany).

Electrophoresis detection of nucleic acids in purified BEVs

To assess the presence of nucleic acids in purified bacterial extracellular vesicles (BEVs), intact BEV suspensions from *E. cloacae* SEA01 and *E. cloacae* C2 strains (see *Materials and Methods*) were analyzed by agarose gel electrophoresis. A total of 10 µL of each BEV sample was mixed with 2 µL of 1× GelRed™ (prepared by diluting 10 µL of a 10,000× stock solution in 1990 µL of Milli-Q water) and 10 µL of 3× loading buffer (0.125% bromophenol blue, 0.25% xylene cyanol, and 15% glycerol in Milli-Q water). The samples waere loaded directly onto a 1% agarose gel prepared with 0.5× TBE buffer and electrophoresed at 80 V for 40 minutes. Nucleic acids were visualized under UV illumination using a Gel Doc

Imaging System (UVP ChemStudio, Analytik Jena; Upland California, USA) equipped with image software.

Preparation of Agarose Gel and TBE buffer

A 1% (w/v) agarose gel was prepared by dissolving 0.3 g of agarose (IBI Scientific, Iowa, USA) in 30 mL of 0.5× TBE buffer. The mixture was heated in a microwave until fully melted, then poured into a gel tray fitted with a comb and allowed to polymerize at room temperature. Once solidified, the comb was carefully removed, and the gel was transferred to an electrophoresis chamber for use. The 5× TBE buffer stock was prepared by dissolving 45 g of Trizma® base and 27.5 g of boric acid in 980 mL of Milli-Q water to obtain the 0.5 × working concentration used in gel preparation and electrophoresis.

Result

Agarose Gel Electrophoresis Confirms DNA Encapsulation in OMVs of *E. cloacae* Strains SEA01 and C2.

Agarose gel electrophoresis confirmed the presence of DNA in the outer membrane vesicles (OMVs) of *Enterobacter cloacae* strains SEA01 and C2. Chromosomal DNA extracted from vegetative cells of both strains (lanes 2 and 6) appeared as high molecular weight bands (>20 kb), with slight retention in the wells, consistent with intact genomic DNA, . In contrast, purified intact OMVs from SEA01 (lane 3) showed strong retention in the wells and faint smearing between 7–10 kb, suggesting the presence of large DNA complexes likely associated with lipids, proteins, or vesicle membranes. OMVs from C2 (lane 7) exhibited a sharper DNA band within the 7 - 10 kb range and reduced retention, indicative of partial vesicle disruption or the presence of smaller, more mobile DNA fragments. No signs of degradation was observed The differing migration patterns between the two strains suggest potential differences in OMV architecture or DNA packaging mechanisms. These results support the hypothesis that OMVs can encapsulate DNA, which may contribute to intercellular communication, genetic exchange, or nutrient provision within biofilm [4, 23,88].

In conclusion, SEA01 OMVs predominantly harbor larger DNA–lipid complexes with reduced mobility, whereas C2 OMVs contain smaller DNA fragments that migrate more freely. This observed heterogeneity may reflect strain-specific strategies of vesicle formation and nucleic acid packaging, warranting further investigation.

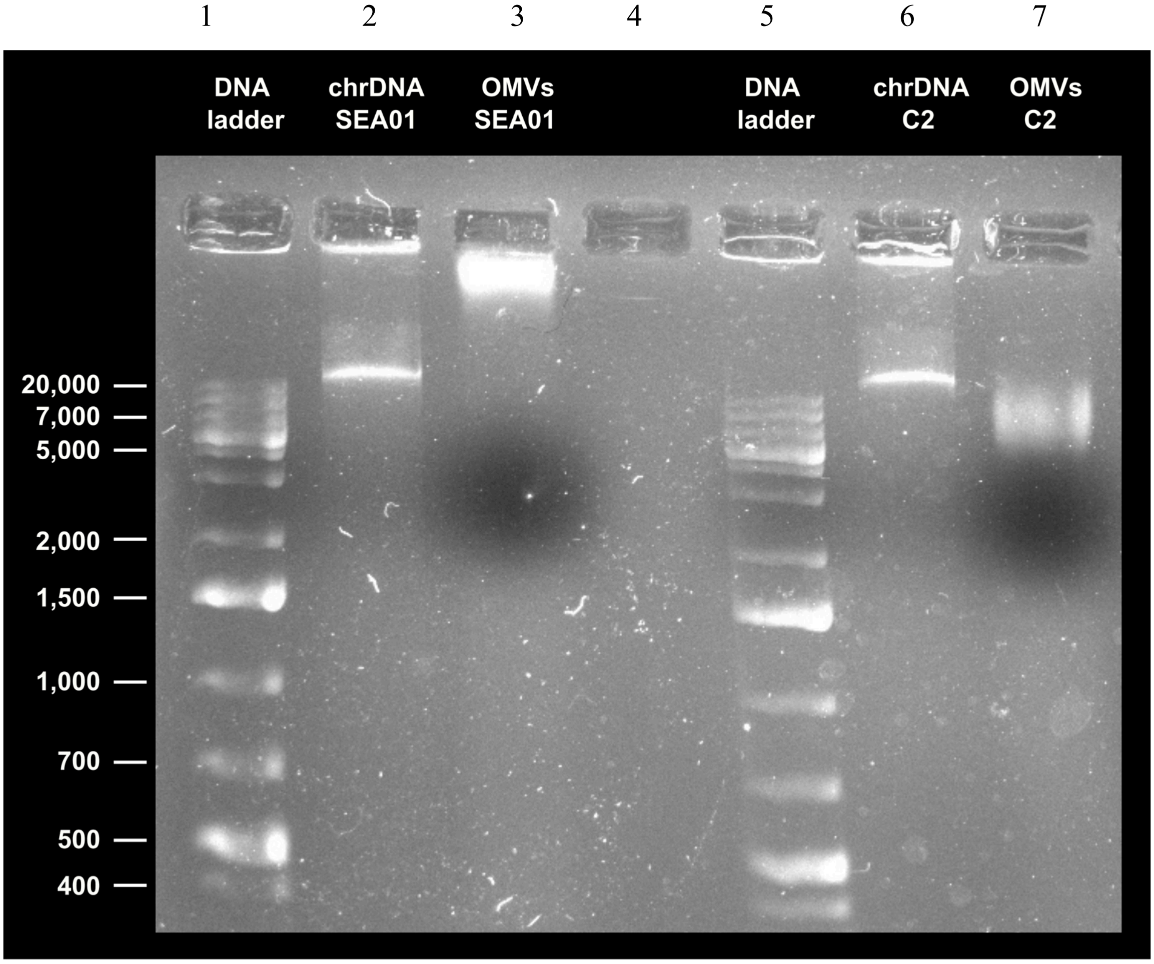

Figure S1. Detection of DNA associated with outer membrane vesicles (OMVs) from *Enterobacter cloacae* strains SEA01 and C2. Agarose gel electrophoresis (1%) illustrating the presence of chromosomal and vesicle-associated DNA. Lane 1 and 5: DNA molecular weight marker. Lane 2: chromosomal DNA extrated from SEA01 (>20 kbp). Lane 3: DNA associated with intact OMVs from SEA01, showing strong retention in the well and faint smearing, indicating large DNA–membrane complexes. Lane 6: chromosomal DNA from strain C2 vegetative cells. Lane 7: DNA associated with OMVs from C2, showing a distinct

band between 7-10 kb. Chromosomal DNA was extracted from 6 h bacterial cultures; while OMVs were analyzed directly without prior DNA extraction.

2. **Detection of Catalase Absence in *E. cloacae* SEA01 by Non-Denaturing Gel Electrophoresis and In-Gel Activity Staining, According to the method of Wayne and Diaz [46].**

Catalase activity was evaluated using native polyacrylamide gel electrophoresis (native PAGE), following the protocol described by Wayne and Diaz [40]. Bacterial strains *E. cloacae* SEA01 and *Bacillus tequilensis* strain 10 were cultured for 12 hours in Tryptic Soy Broth (TSB), 32°C and harvested by centrifugation. Cells were lysed mechanically using sterile glass beads in phosphate buffer (pH 7.8) with PMSF 0.2M as protease inhibitor. Protein concentration was determined using the Bradford method, and 10 µg of total soluble protein from each strain was loaded onto an 8% native PAGE gel. After electrophoresis, gels were incubated with a staining solution containing hydrogen peroxide by 20 min and then potassium ferricyanide/ferric chloride to detect catalase activity. Active catalase appeared as achromatic (clear) bands due to the degradation of hydrogen peroxide, preventing Prussian blue formation.

Result: The absence of a detectable catalase band in *E. cloacae* SEA01 confirms previous findings of its catalase-negative phenotype observed in plate-based enzymatic assays. This enzymatic deficiency may reflect an adaptive strategy associated with enhanced outer membrane vesicle (OMV) production and biofilm formation. This potentially facilitating the mitigation of host-derived reactive oxygen species (ROS) during plant interaction and colonization. Such a mechanism may be particularly advantageous in the agave rhizosphere, where plants are frequently exposed to environmental stress and release ROS as part of their innate immune response against microbial interaction.

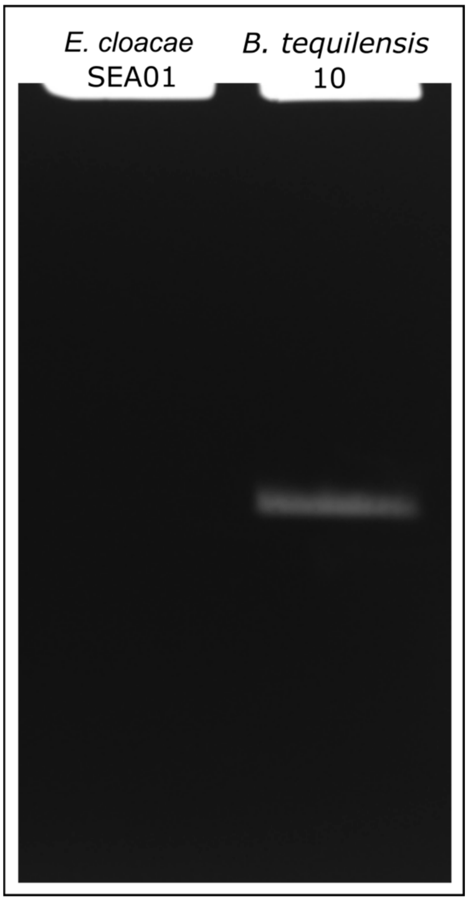

Figure S2. Representative native PAGE gel showing in-gel catalase activity. An 8% native polyacrylamide gel was loaded with 10 µg of soluble proteins extracted from *E. cloacae* SEA01 and *Bacillus tequilensis* strain 10. Catalase activity was visualized as achromatic (clear) bands resulting from competition for hydrogen peroxide between the enzyme and Prussian blue staining solution [46]. Notably, *E. cloacae* SEA01 lacks a detectable catalase activity band, whereas *B. tequilensis* exhibits a clear band consistent with their catalase activity.

3. Screening the Growth-Promoting Effect of Bacteria on *Agave* plantlets

A) Plant Acclimatization in Microcosms

Micropropagated plants of *Agave tequilana* were used for growth promotion experiments. Plantlets were cultivated in microcosms consisting of glass bottles containing 100 g of autoclaved beach sand. The plants were watered every two weeks for one month with 25 ml of sterile distilled water to maintain a relative humidity of 50 % in a growth chamber (Climacell) set at 28 °C, with a 14 h/10 h light/dark photoperiod and a light intensity of 400 µmol m^{-2} s^{-1}. The beach sand was collected from "Punta Perula" beach in La Huerta, Jalisco, Mexico (latitude: 19.587855, longitude: 105.128493), washed extensively with tap water, air-dried and autoclaved for 3 hours prior to use.

B) Plant Inoculation

Enterobacter cloacae SEA01 was cultured in tryptic soy broth (TSB) at 32°C for 16 hours to mid-logarithmic phase, centrifuged at 6000 rpm for 10 minutes and washed three times with sterile 0.05% sucrose solution. The bacterial suspension was adjusted to an OD_{600} of 0.2 (equivalent to 5.3×10^5 CFU mL^{-1}). A six-month trial was conducted in which 10 mL of the suspension was applied to each plantlet every month. The control plants received only a 0.05% sucrose solution. Each treatment comprised 10 plants. After harvesting, the roots were rinsed to remove sand and the dry biomass per plant was measured.

Results:

Agave plants are mainly exposed to conditions of low nutrient availability, particularly limited access to nitrogen and phosphorus, combined with extreme environmental conditions. Due to this, strain SEA01 was selected for its nitrogen fixation capability, phosphate solubilization, and ACC deaminase activity. In addition, previous studies highlight the protective and biostimulant properties of this bacterial genus [51-53]. Figure shows the biometric differences between *A. tequilana* plants treated with *Enterobacter cloacae* SEA01 and control plants irrigated with 0.05% sucrose in nutrient-free beach sand over six months. Plants inoculated with the endophyte exhibited significantly larger leaves ("antennae") and

more developed root systems. The leaf length in treated plants was approximately twice that of controls and displayed characteristic green-reddish pigmentation. No significant difference was observed in the number of newly formed leaves. The root systems of the treated plants showed increased root number, length, and altered morphology, with microscopic analysis revealing changes in root thickness and cellular organization (Figure 2A). Total dry biomass was higher in *E. cloacae* SEA01–treated seedlings (*1.62 ± 0.08 g*, n = 10) than in water controls (*0.83 ± 0.06 g*, n = 10), a ~96% increase; Student's t-test: $t(18) = 24.51$, $p < 0.0001$. Because there are only two groups, the t-test is equivalent to one-way ANOVA ($F(1,18) = t^2$).

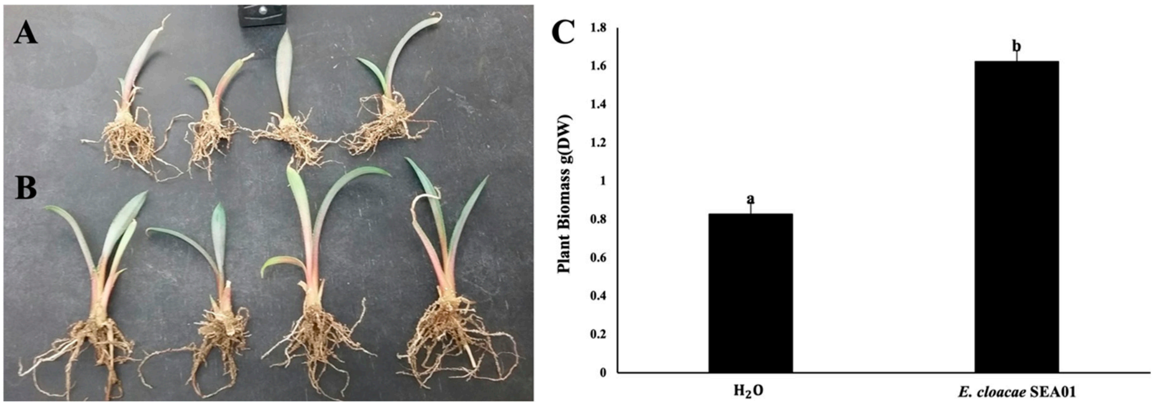

Figure S3. Effect of *E. cloacae* SEA01 on the total dry biomass of *A. tequilana* plantlets after six months of treatment. **A.** Plants irrigated monthly with water-sucrose 0.05% solution (control). **B.** *E. cloacae* SEA01 cells suspended in 0.05% sucrose. **C.** Data are mean ± SD (n = 10 per group). Different letters indicate statistical significance ($p \leq 0.05$).

4. **PCA analysis of Lipidome data.**

The lipidomics analysis of OVMs was performed in an exploratory approach with two replicates due to the technical challenges of retrieving enough lipidic material from the OMVs and MS data of high quality with high sensitivity. The results obtained by lipidomics analysis of the two replicates revealed important trends in alteration of the percentage composition of lipid classes in the OMVs compared to the composition of the bacterial membrane. The alterations observed provide evidence and trends that can guide further investigations. We have performed a Biplot Principal Component Analysis (PCA) analysis of the lipidomics data, which was included in the Supplementary data reinforcing the observed trend in variation of the OMVs lipids relative percentage composition. The PCA analysis considered the whole dataset of lipids identified in the OMVs and revealed that most of the variance of the data could be explained by the difference between Vesicles and Bacterial membrane lipid percentage composition.

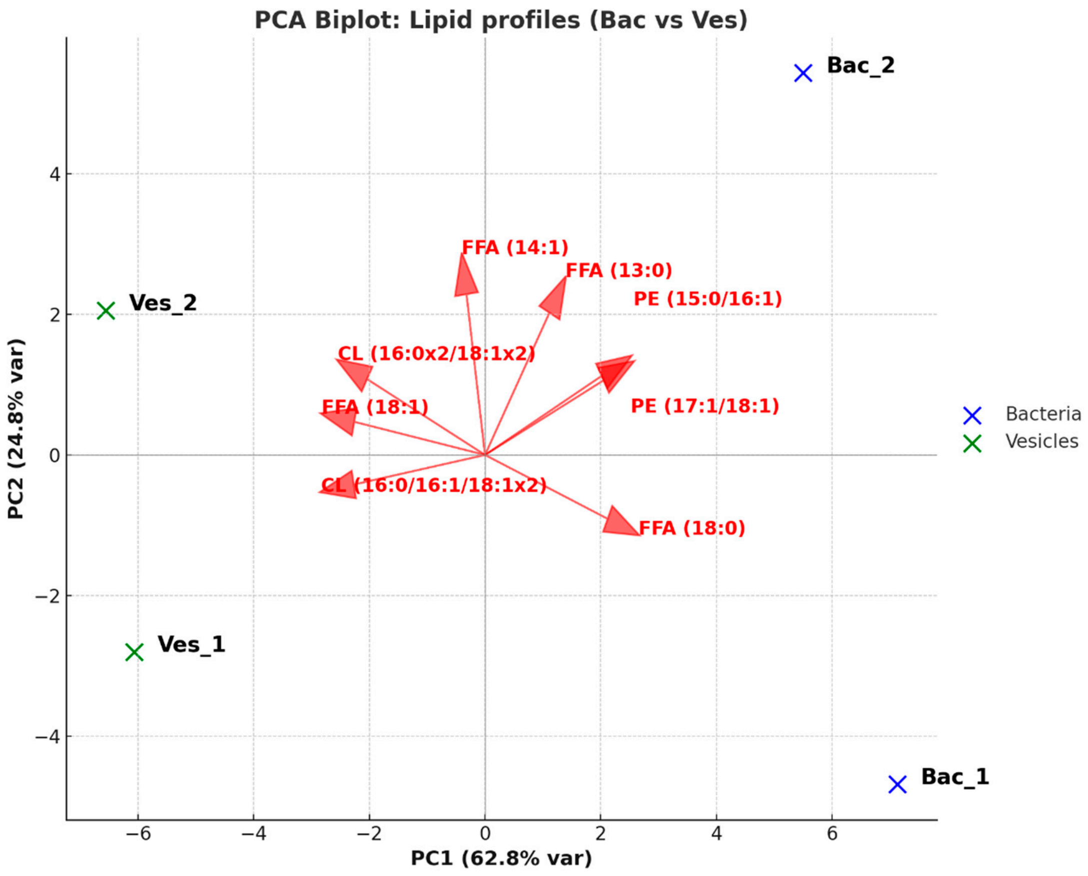

Figure S4. Principal component analysis of lipidomics data from whole Bacteria and bacteria´s Outer membrane vesicles. Relative percentage of lipids composition was calculated based on Mass spectrometry-based analysis of lipidomics from whole Bacteria (Bac) and Outer membrane vesicles (Ves) and data analyzed was performed by Principal Component Analysis. The lipids which contribute most to the data dissimilarity are indicated by arrows in the PCA Biplot. (N=2).
