# Supplementary figures and images for: Outer Membrane Vesicles, Lipidome, and Biofilm Formation in the Endophyte Enterobacter Cloacae SEA01 from Agave Tequilana
Source: Microorganisms. 2025 Oct 23;13(11):2432. doi: 10.3390/microorganisms13112432 (PMC12654736; doi:10.3390/microorganisms13112432)

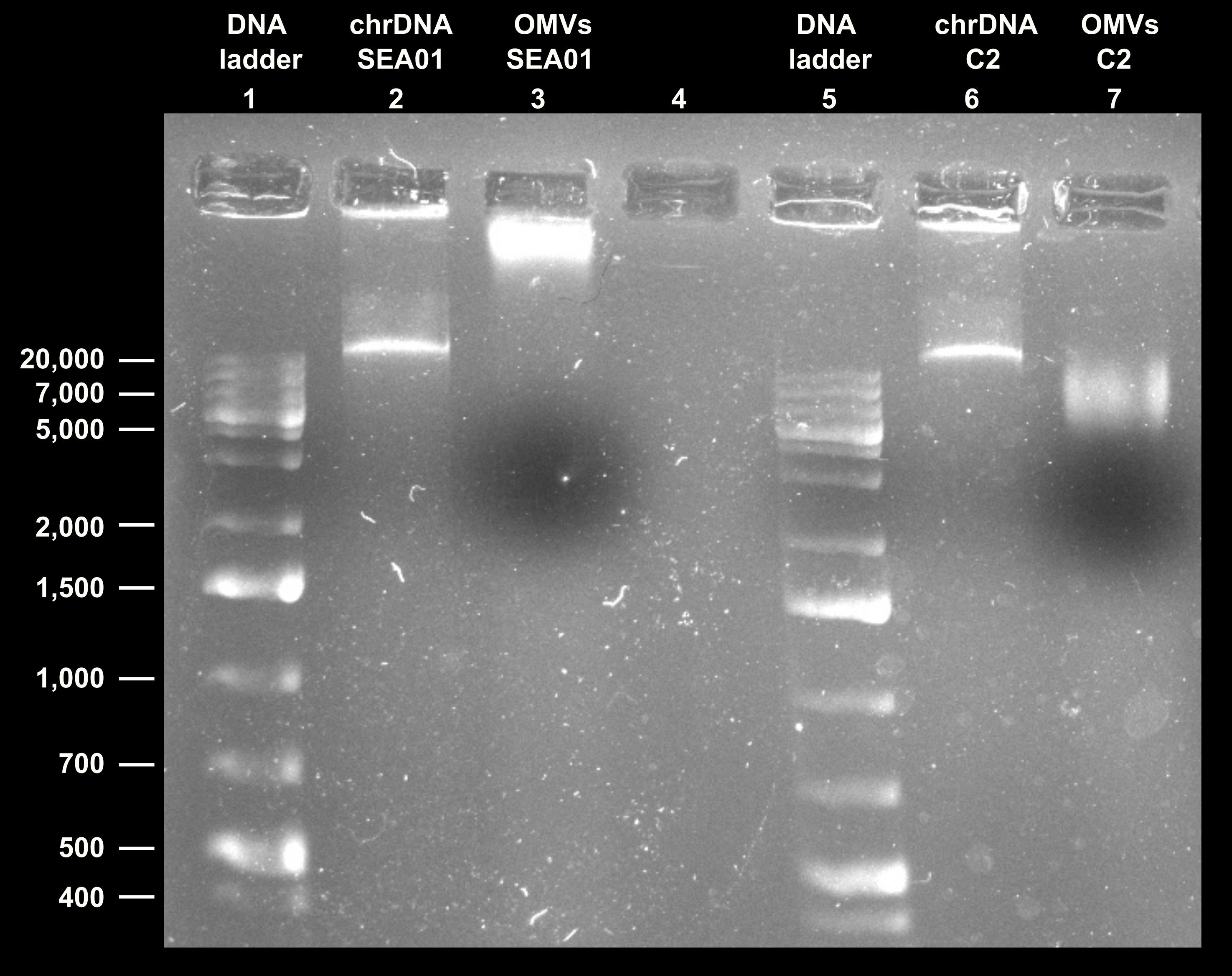

Supplement: Supplementary file 1 [file microorganisms-13-02432-s001.zip › Fig. S1.tiff]

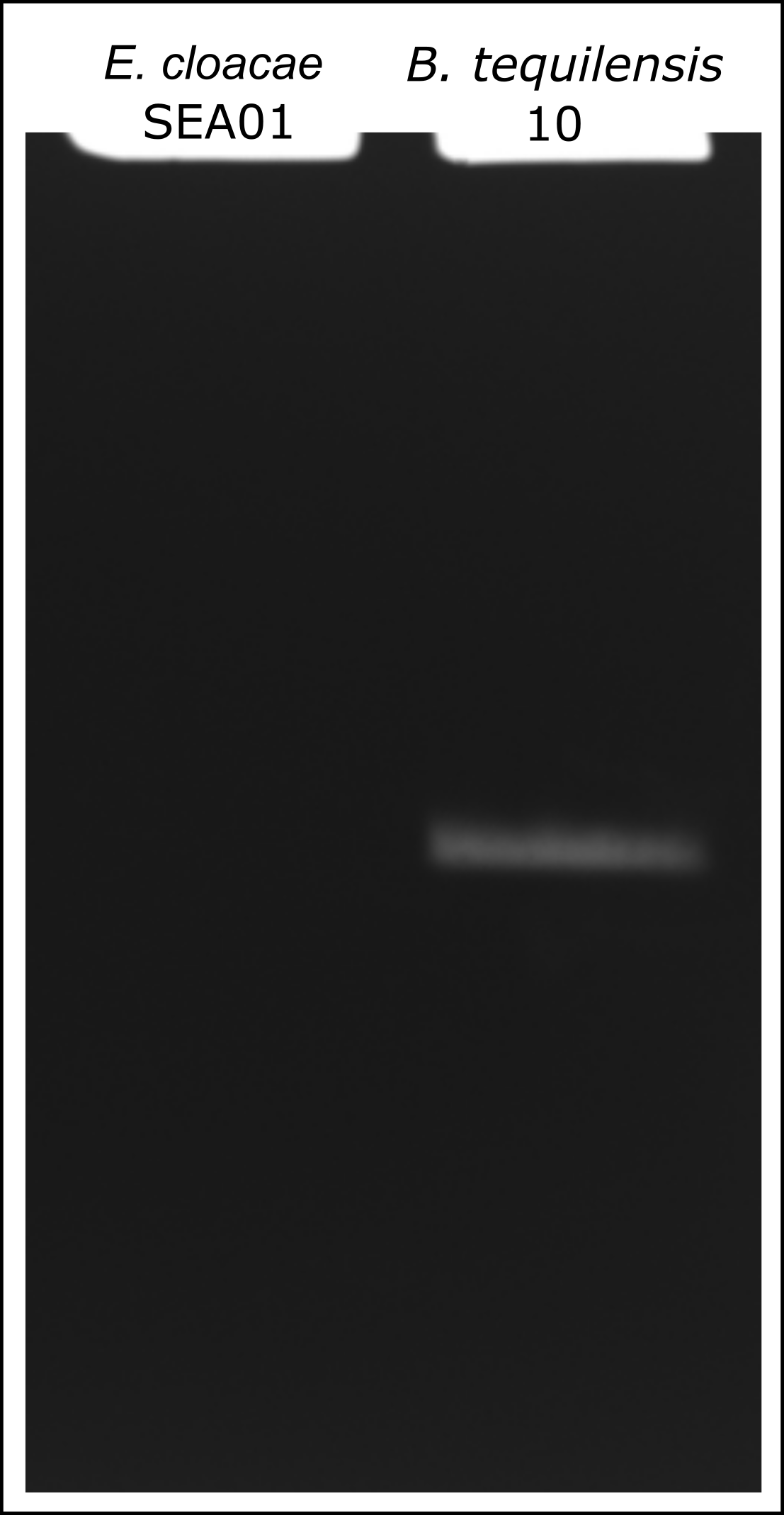

Supplement: Supplementary file 1 [file microorganisms-13-02432-s001.zip › Fig. S2.png]

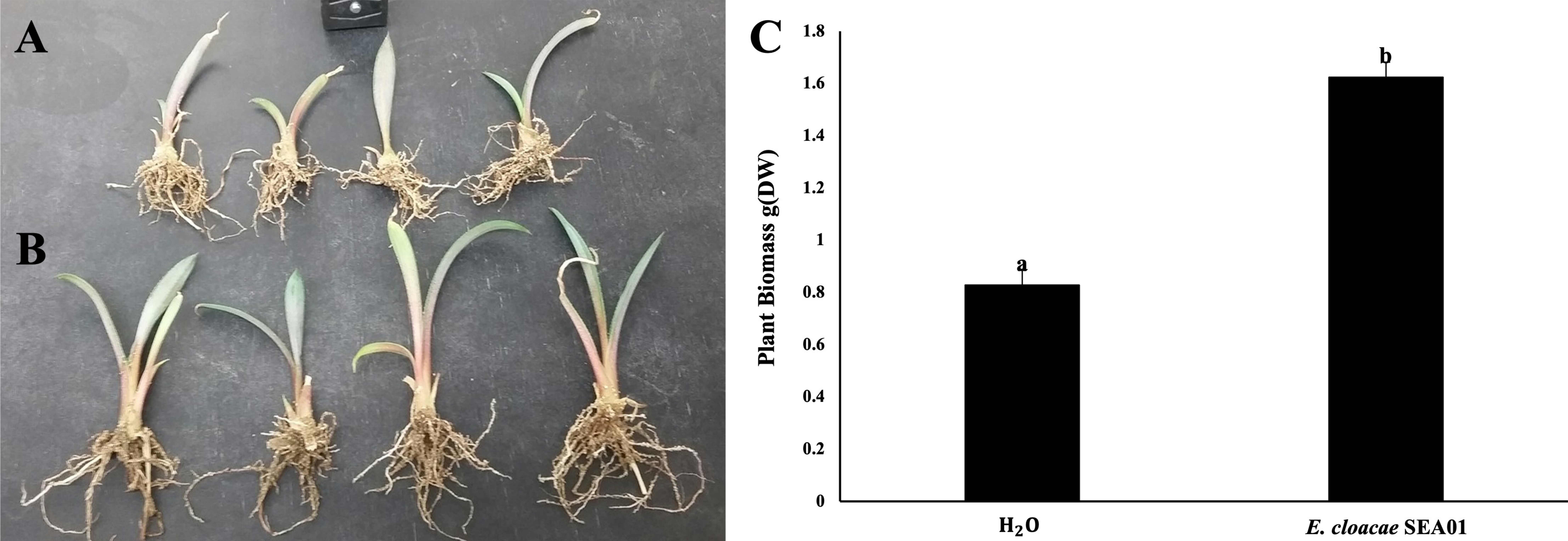

Supplement: Supplementary file 1 [file microorganisms-13-02432-s001.zip › Fig. S3.tiff]

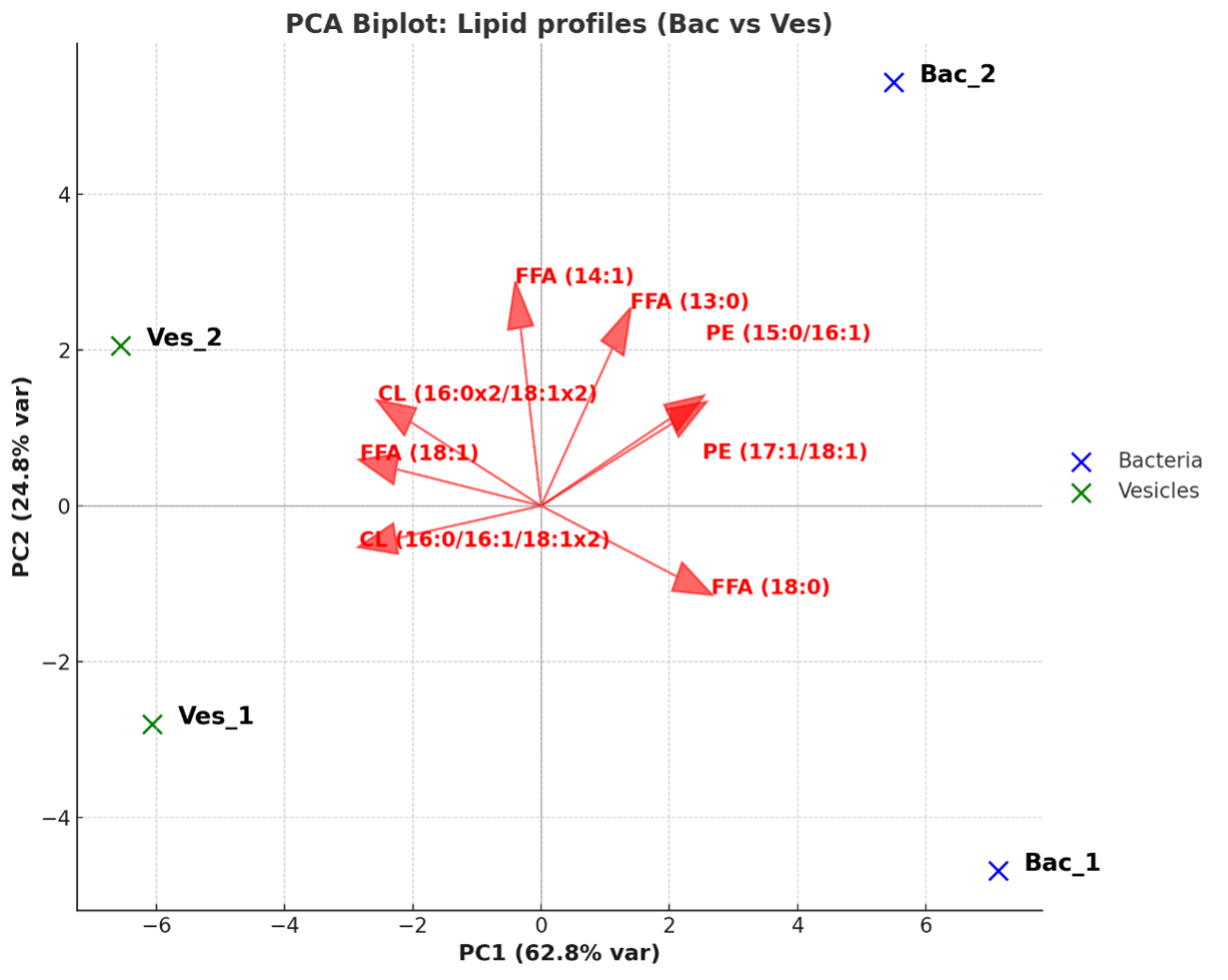

Supplement: Supplementary file 1 [file microorganisms-13-02432-s001.zip › Fig. S4.png]
